# Anatomical, Physiological, and Transcriptome Analyses Revealing Pod Shattering of *Medicago ruthenica* Associated with Pericarp Lignin Biosynthesis

**DOI:** 10.3390/biom15091269

**Published:** 2025-09-02

**Authors:** Lin Zhu, Maowei Guo, Zhiyong Li, Jun Li, Hongyan Li, Zinian Wu, Yonglei Tian, Chenggui Zhao

**Affiliations:** 1 Institute of Grassland Research, Chinese Academy of Agricultural Sciences, Hohhot 010013, China; zhuhl1413@126.com (L.Z.);; 2 Key Laboratory of Grassland Resources and Utilization of Ministry of Agriculture, Hohhot 010013, China; 3 Key Laboratory of Herbage & Endemic Crop Biology, Ministry of Education, School of Life Sciences, Inner Mongolia University, Hohhot 010021, China; 4 Bureau of Agricultural and Animal Husbandry Technology of Liangcheng County, Wulanchabu 013750, China

**Keywords:** *Medicago ruthenica*, pod shattering, lignified endocarp, phenylpropanoid biosynthesis, auxin

## Abstract

**Background**: *Medicago ruthenica*, a perennial legume forage valuable for ecological restoration and improved breeding, suffers significant harvest losses due to pod shattering. Pod shattering is a trait not only linked to not only pod ventral suture, but also pericarp properties. In this study, we aimed to (1) elucidate the role of pericarp in explosive pod shattering by comparing shattering-susceptible (SPD) and shattering-resistant (RPD) *M. ruthenica* genotypes, and (2) identify key regulatory genes and pathways underlying this mechanism. **Methods**: We conducted comparative analyses of pericarp anatomy and physiological traits (pericarp components such as water content, cellulose, hemicellulose, pectin, and lignin; and the activities of enzymes such as cellulose synthase A (CesA), phenylalanine ammonia-lyase (PAL), 4-coumarate: CoA ligase (4CL), cinnamyl alcohol dehydrogenase (CAD), and peroxidase (POD) in SPD and RPD pods). Transcriptome of pod pericarps identified differentially expressed genes (DEGs) for the selection of candidates functional genes. Promoter analysis was performed on candidate functional genes to identify specific regulated factors. The functional role of auxin signaling was validated through exogenous auxin application and the assessment of pod shattering rates and gene expression. **Results**: SPD pod pericarps exhibited significantly higher lignification of endocarp, lignin, cellulose, hemicellulose and pectin content, but lower water content than RPD. Principal component analysis identified that lignin contributes the highest loading value (0.727) contributor to pod shattering. The activities of five cell wall biosynthesis enzymes were higher in SPD pod pericarps than RPD. Transcriptome analysis identified more than 3419 DEGs in SPD pericarps. KEGG enrichment highlighted “phenylpropanoid biosynthesis” as the most significant pathway. A total of 57 lignin-biosynthesis-related DEGs were upregulated in SPD, including 15 *POD*s. Promoters of 11 *POD* genes contained *MYB*-binding motifs and 8 contained auxin-responsive elements, a total of 76 *MYB* transcription factors (mostly upregulated) and 9 auxin biosynthesis genes (mostly downregulated) were differentially expressed in SPD. Exogenous auxin application significantly reduced SPD pod shattering to 23.6% and concurrently downregulated *POD*s expression. **Conclusions**: This study establishes that enhanced lignification within the pericarp endocarp by the upregulation of lignin biosynthetic genes (particularly *POD*s), coupled with upregulation by *MYB* transcription factors and downregulation by auxin, is a core mechanism of explosive pod shattering in *M. ruthenica*. The identified DEGs, especially *MYB*s, *POD*s, and auxin pathway genes, provide gene information for breeding shattering-resistant *M. ruthenica* varieties through molecular design or marker-assisted selection.

## 1. Introduction

*Medicago ruthenica*, a perennial legume-forage species found across the steppes of Eurasia [1,2], has emerged as a valuable and preferred grass in China. Owing to its high biotic and abiotic stress resistance compared to other *Medicago* species, *M. ruthenica* has been used as forage in areas that frequently experience drought and extreme cold; additionally, the species is characterized by wide genetic diversity and abundant gene resources for improving environmental stress tolerance of alfalfa [3,4,5]. Furthermore, it has become increasingly important owing to its restoration service, which is more efficient than that of *Medicago* and other legume grass species [6]. *M. ruthenica* has considerable potential for applications in the ecological restoration of degraded grasslands and the establishment of artificial grasslands, where there is a high demand for seed.

Seed yield is determined by the number of pods, number of seeds per pod, one-thousand-seed weight, and pod shattering rate. In particular, pod shattering is unfavorable for agricultural production because it results in a low seed yield and leads to severe weed problems in subsequent crop rotations [7]. Furthermore, intense pod shattering in *M. ruthenica* (including wild accessions and all bred cultivars) leads to severe seed yield losses of up to 80% at harvest [8]. Furthermore, because of low synchrony in pod maturation due to the indeterminate flowering pattern of the species, farmers often collect a mass of immature seeds when harvest is performed prematurely, and a large number of seeds fall on the ground during harvesting, such that farmers have to use ground-film treatment and pick up fallen seeds during harvest, which requires additional manual processes that increase production costs. Thus, breeding and cropping cultivars with shattering-resistant traits suitable for mechanization represent an optimal potential solution for *M. ruthenica* seed production.

In recent decades, extensive research has revealed the morphological, anatomical, and genetic factors that regulate pod shattering, which is typically initiated with shattering along the ventral and dorsal sutures, followed by spiral twisting of pod walls. Pod shattering results from gene mutations, deletions, and overexpression that lead to abnormal development of pod ventral suture or pod shattering traits. Thus, for example, abnormal lignified fiber cap cells (FCCs), caused by *SHAT1-5* overexpression in the abscission zone, are the determining factor for the pod shatter-resistant phenotype in soybean (*Glycine max*) [9]. Meanwhile, defects in Pdh1 include a premature stop codon that cannot promote pod shattering by torsion of the dried pod valves or create sufficient driving force [10]. In turn, *SHP*/*FUL*/*IND* expression leads to pod shattering in *Arabidopsis* and *Brassica napus* by promoting or inhibiting pod valve-margin lignification [11,12,13]. Apart from the aforementioned factors, the anatomical structure and chemical components of pod pericarps, such as the hemicellulose, cellulose, and lignin contents, were also correlated with susceptibility to shattering [10,14].

**Figure 1:**
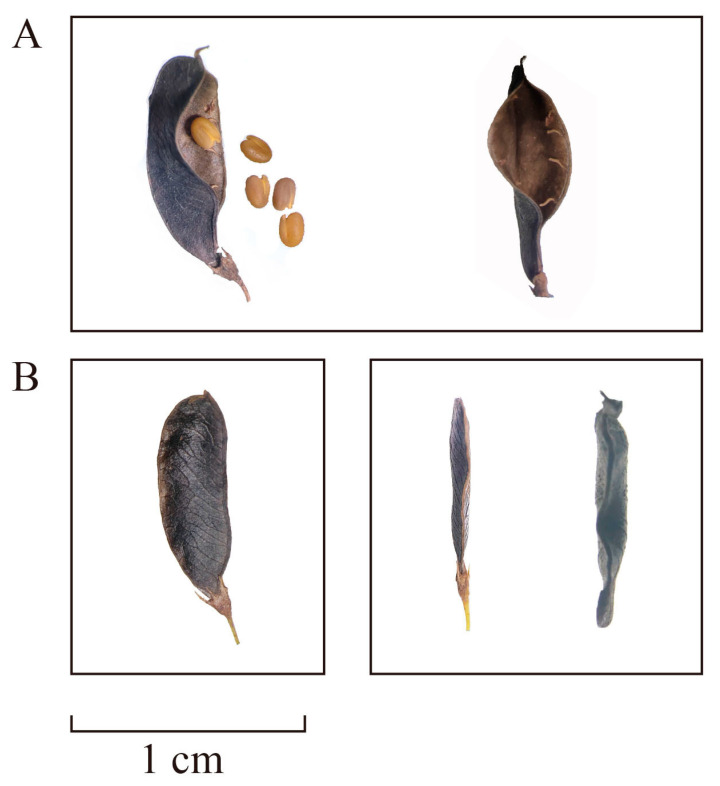
Mature pods of *M. ruthenica*. Photographs of pods were captured using a stereo microscope (XTL-3000C). Picture (**A**) shows pods of the pod shattering-susceptible genotype (SPD), including a side view (**left**) and front view (**right**) of fully opened pods. Picture (**B**) shows pods of the pod shattering-resistant genotype (RPD), including a side view (**left**) and front view of a fully closed pod (**middle**) and a front view of an incompletely opened pod (**right**). In the incompletely opened pod, the abscission zone in the ventral suture is destroyed; however, the fissure prevents seeds from dropping out of the pod.

Over years of natural germplasm collection and field breeding, our research group has obtained a pod shattering-resistant genotype (RPD) with a pod shattering rate lower than 5% [8,15], which can be compared with a pod shattering-susceptible genotype (SPD) with a pod shattering rate greater than 76%. We found that previously reported shattering-related genes are unsuitable for interpreting the mechanism of pod shattering in *M. ruthenica*. Firstly, barely any differences in the expression of homologous *Pdh*1 and *IND* genes were observed between SPD and RPD [15,16,17,18], a phenomenon that also occurs with the SHP and FUL genes [8]. Secondly, in contrast to the coiled pods of alfalfa, a GCA site in the *SHP* gene of *M. ruthenica* results in a straight-pod morphology [19], implying that the mature pericarp does not twist spirally during pod shattering, unlike in soybean, *Vicia sativa*, and *Lotus corniculatus*. Thirdly, abnormal lignification of FCCs in the abscission zone was not observed in RPD pods [8]. More importantly, three types of pod shattering morphologies occur in *M. ruthenica*: wholly opening pods that allow seed shedding, wholly closed pods that do not allow seed shedding, and incompletely opening pods that do not allow seed shedding (Figure 1). Although several pod shattering-related genes were identified in our previous study, the function of these genes—encoding polygalacturonase and cellulase—can only explain cell degradation in the abscission zone [8], which leads to complete pod opening rather than incomplete pod opening, despite clear abscission zone destruction in the latter.

To date, the mechanisms of the three kinds of pod shattering morphology are not well understood, and little is known about the effect of pod pericarp function on pod shattering in *M. ruthenica*. Cell wall components of pod pericarp appear to be linked to the strength of fruit shattering [18,20,21,22,23]. Here, we aimed to systematically elucidate the intrinsic differences using pod shattering-susceptible (SPD) and shattering-resistant (RPD) *M. ruthenica* genotypes: comparative anatomical studies identified key phenotypic differences in pericarp structure; physiological and biochemical profiling elucidated the material basis and underlying processes driving these structural variations; and transcriptomic comparisons revealed the specific functional genes responsible for the observed difference. These methods were combined with promoter analyses to reveal the potential upstream regulatory factors and hormonal signaling underlying the mechanism of explosive pod shattering in *M. ruthenica*.

## 2. Materials and Methods

### 2.1. Plant Materials

The SPD and RPD genotypes were supplied by the Institute of Grassland Research of the Chinese Academy of Agricultural Sciences. The two genotypes were screened by evaluating the pod shattering rate over three consecutive years (2016–2019) among 271 *M. ruthenica* accessions obtained from the National Medium-term Gene Bank of Forage Germplasm in Hohhot, China.

Seeds of both genotypes were first germinated in a greenhouse and planted in a randomized block design once the seedlings had three fully developed leaves; finally, they were transplanted at the SharaQin Key Wild Scientific Monitoring Station of the Academy of Agricultural Sciences of China. Sixteen individual plants of each selected accession were planted. The experimental field plots were managed using an automatic irrigation system and routine farming methods. The mean annual temperature at the study site is 5.8 °C, and the mean annual precipitation is 300 mm. After preliminary field screening, we filtered 50 accessions and determined the pod shattering rate in each case. The trait showed a normal distribution [15]. Based on high similarity of growth period and plant phenotype, but with significantly different pod shattering rates, the two genotypes used in this study were obtained from the same accession among the 50 accessions evaluated. Three biological replicates were used of each genotype [8].

### 2.2. Sampling Preparation

Before sampling, inflorescences were marked. Only florets with similar development were retained (the standard exceeds the calyx by approximately 4–5 mm; the standard and wing petals of the papilionaceous flower open, while the stigma and stamens do not emerge from the keel petal), identified using colored strings tied around the pedicels. During the sampling process, inflorescences were continuously marked, because *M. ruthenica* is an indefinite inflorescence forage. Anatomical analyses were performed on samples obtained at 4-day intervals, from 4 to 36 days after flowering (DAF). At each sampling timepoint, whole pods were collected for anatomical analysis. Ten pods were randomly collected from each biological replicate of each genotype at each sampling timepoint, and they were paraffin-embedded and sectioned; ten pods were collected randomly from each biological replicate of each genotype at each sampling timepoint for transmission electron microscopy. Water content was determined in pod pericarp samples (pods without seeds) obtained from 8 to 32 DAF at 4-day intervals. Pod samples at 8, 12, 16, and 20 DAF and pods without seeds, ventral suture, and dorsal suture (i.e., only pod pericarp) were collected for physiological and molecular analyses.

### 2.3. Anatomical Profile of Pod Pericarps

Pod samples were randomly collected in the field and immediately fixed in FAA solution (90:5:5, 70% ethanol/formalin/acetic acid) after removing seeds with an anatomical needle. The samples were then paraffin-embedded and sectioned with a thickness of 10–20 µm using an AO Spencer 820 rotary microtome (Ramsey, MN, USA). These sections were placed in the safranin O staining solution to visualize the pod pericarp structure for 3–5 s and then decolorized by immersing in three cylinders containing 50%, 70%, and 80% alcohol for 3–8 s. This was followed by staining in fast green solution for 4–6 s and anhydrous ethanol three-cylinder dehydration. The sections were then transparentized in xylene for 5 min, and the tissue section was mounted with neutral gum. The abovementioned reagents were supplied by Servicebio Co., Ltd. (Wuhan, China). The sections were observed under an Eclipse E100 optical microscope (Nikon Corp, Tokyo, Japan). The lignified cell walls appeared red, and the cell walls lacking lignin appeared green. Three biological and technical replicates from the two genotypes were used for each sample and for the different sampling timepoints.

For transmission electron microscopy (TEM) of pod pericarp cells, pod samples were immediately placed in TEM fixative (Servicebio Co., Ltd., Wuhan, China, G1102) after sampling. In addition, pod samples of 16 days after flowering (DAF) were collected for scanning electron microscope (SEM) observation. These samples were collected and immediately put into the electron microscope fixative (Servicebio Co., Ltd., Wuhan, China, G1102-10ML) for 2 h. Samples were then fixed with 1% OsO4 (Ted Pella Inc., 18456) for 7 h in the dark, followed by rinsing. Subsequently, samples were sequentially dehydrated in an ascending series of ethanol solutions (30%, 50%, 70%, 80%, 90%, and 100%, Sinaopharm Group Chemical Reagent Co., Ltd., Shanghai, China, 100092183) for 1 h per step at room temperature (25 °C). This was followed by dehydration with ethanol/acetone (Sinaopharm Group Chemical Reagent Co., Ltd., 10000418, 3:1) for 0.5 h, ethanol/acetone (1:1) for 0.5 h, ethanol/acetone (1:3) for 0.5 h, and acetone for 1 h. Additionally, resin penetration and embedding were performed. Samples were then placed in an oven at 60 °C for approximately 48 h to allow polymerization and then cut into 60–80 nm thin sections using an ultra-microtome (Leica, Wetzlar, Germany, Leica EM UC7). The sections were stained in a 2% uranium acetate-saturated alcohol solution (SPI, 90529-77-4) for 8 min in the dark and then rinsed thrice with 70% ethanol and ultra-pure water. Next, the sections were stained thrice with a 2.6% lead citrate solution (Sinaopharm Group Chemical Reagent Co., Ltd., 10019408) for 8 min to avoid CO_2_ and then rinsed with ultra-pure water. After overnight drying, sections were observed using TEM (Hitachi, Japan, HT7800/HT7700), and images were captured. Cell wall thickness and lumen diameter of endocarp measurement using ImageJ (FITS, 2025), thirty sclereid were randomly selected in the visual scale of 20 μm, each cell was measured in several directions at least 4 times owing to its irregularity morphology.

### 2.4. Component Analysis and Related Synthetase Activities

Pod pericarp samples used for water content analysis were collected in the field. Seeds were removed using an anatomical needle, placed on dry ice, and transported to the laboratory. Then, 2000 g of fresh samples were weighed, placed into an envelope, and dried at 65 °C to constant mass prior to measuring the dry weight. Each genotype was represented by three biological replicates, and each biological replicate was used for three technical replicates during each stage (a total of seven stages). Water content was calculated as follows: water content% = (fresh weight − dry weight)/fresh weight.

Pod pericarps collected at 8, 12, 16, and 20 DAF were used to determine the cellulose, hemicellulose, total pectin, and lignin contents by using 0.1 g fresh samples [24,25]. Three biological replicates were used for each genotype at each stage, and three technical replicates were used for each biological replicate.

The activities of cellulose synthase A, (CesA), and four lignin synthase enzymes (phenylalanine ammonia-lyase, PAL; 4-coumarate:CoA ligase, 4CL; cinnamyl-alcohol dehydrogenase, CAD; peroxidase, POD) were determined as described by Duan and Li [25,26], respectively. Each index was determined using a 0.1 g fresh sample. The seeds of samples were removed by an anatomical needle, and ventral sutures and dorsal sutures were manually removed using surgical scissors; then, samples were immediately frozen in liquid nitrogen and stored at −80 °C until use. Enzymes were extracted using PBS buffer. The OD of the supernatant was measured at a wavelength of 450 nm for CesA, 290 nm for PAL, 333 nm for 4CL, 450 nm for CAD, and 470 nm for POD. Three biological replicates and three technical replicates were included per sample.

### 2.5. RNA Extraction and Transcriptomic Analysis

Total RNA was extracted from pod pericarps using the TRIzol reagent (Invitrogen, Carlsbad, CA, USA) according to manufacturer instructions. Sampling of pod pericarps was performed as described above. Methods were followed for total RNA purity and quality evaluation, transcriptome sequencing [8], reference genome matching [27], differentially expressed gene (DEG) analysis, and gene function annotations. Raw sequence data were deposited and are available from the NCBI database under accession number: SUB9998822.

Fragments per kilobase of transcript per million fragments mapped (FPKM) were used for DEG analysis to calculate the gene expression using the maximum flow algorithm. DEG analysis was performed using DESeq2 by threshold value of false discovery rate (FDR) ≤ 0.05 and fold change value (FC) ≥ 2. The enrichment Gene Ontology (GO) terms and enrichment differential genes among samples were analyzed according to |log_2_ (FC)| ≥ 1 and *p* value < 0.01. RPD samples served as controls. For upregulated genes, expression levels in SPD samples were higher than the corresponding expression levels of the same genes in RPD samples. Conversely, downregulated genes were those whose expression levels in SPD samples were lower than the corresponding levels of expression of the same genes in RPD samples.

### 2.6. Quantitative Real Time-Polymerase Chain Reaction (RT-qPCR) Analysis

Total RNA extraction was performed as described above. Subsequently, first-strand cDNA synthesis was performed according to manufacturer instructions (TIANGEN, Beijing, China). Each PCR was performed for 15 min at 42 °C using a 20 μL reaction mixture and for 3 min at 95 °C; PCR products were stored at −20 °C for RT-qPCR conducted using an Applied Biosystems™ QuantStudio™ 6 real-time PCR system (Applied Biosystems, Foster City, CA, USA). Each PCR was performed in a 20 μL mixture (including 2X SG Fast qPCR Master Mix 10 μL, forward and reverse primer 0.8 μL, DNF buffer 2 μL, cDNA 1 μL, and ddH2O 6.2 μL) for 3 min at 95 °C and for 30 s (40 cycles) at 60 °C. A total of 20 primer pairs for RT-qPCR were designed using the Primer software version 5.0 (Table S9), and synthesized by Sangon Biotech Co., Ltd. (Shanghai, China). Additionally, *EF-1α* was used as the reference gene for normalization. Each sample was analyzed in triplicate. The data were analyzed using the 2^−ΔΔCt^ method [28].

### 2.7. Promoter Motif Analysis

To detect the binding sites of transcription factors (TFs) for genes related to complete pod shattering, the sequences at 2000 bp upstream of annotated translation-start sites from the reference genome were obtained using TBtools v2.083 [27,29]. Promoter motif analysis was performed using the PlantCARE database (http://bioinformatics.psb.ugent.be/webtools/plantcare/html, accessed on 14 March 2022).

### 2.8. Verification of Exogenous Auxin Spraying

To verify whether auxin negatively regulates pod shattering in *M. ruthenica*, we sprayed 4-chlorophenoxy acetic acid (4-CPA, Sigma-Aldrich, Shanghai, China, PH011009), a synthetic auxin widely used as a plant growth regulator in place of the natural auxin IAA, on SPD and RPD. 4-CPA solutions of varying concentrations (0, 50, 100, and 150 mg/L) were sprayed on SPD and RPD at 7 d intervals starting at the initiation of the blooming stage. Water (0 mg/L 4-CPA) was sprayed as control treatment, avoiding the time of pollination. Each treatment was applied in quintuplicate. Pod shattering rates were estimated according to the method described by Guo [8]. Briefly, mature pods were collected from whole plants and divided into three portions. Then, 100 pods were randomly counted within each portion, and finally, the average pod shattering rate was calculated. SPD pod samples for gene expression analysis were collected beginning at the blooming stage, as described in Section 2.2, and RT-qPCR analysis was performed as described in Section 2.5.

### 2.9. Statistical Analysis

Statistical analysis was performed using Excel 2010 (Microsoft, Washington, DC, USA). Differences in physiological and biochemical data were tested using a one-way analysis of variance (ANOVA) and homogeneous subsets analysis in SPSS 17.0 (IBM, Armonk, NY, USA). Figures were generated using the R programming language. Subsequently, figures and column and line charts were created using Origin 2019 (OriginLab, Northampton, MA, USA). Venn plots and heat maps were created using Tbtools. Anatomical illustrations were generated using Adobe Illustrator CC 2018 and Adobe Photoshop CC 2019 (Adobe, San Jose, CA, USA).

## 3. Results

### 3.1. Anatomical Characteristics Associated with Pod Shattering

**Figure 2:**
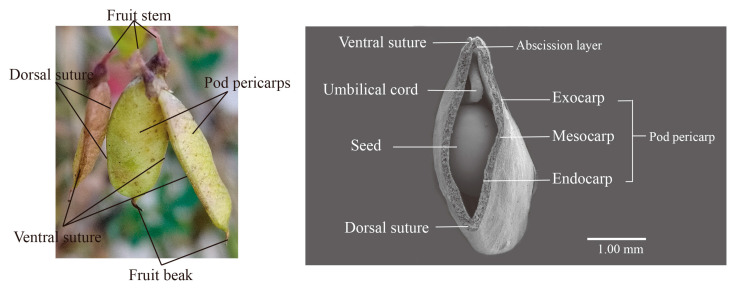
Phenotypic and microscopic morphology of pods. **Left**: Pod morphology of *M. ruthenica* in the field during the yellow maturation stage of the pod. **Right**: Scanning electron microscope (SEM) image of a cross-section of the whole pod at 16 days after flowering (DAF). The pod of *M. ruthenica* is composed of a fruit beak, pod pericarp, and fruit stalk. The pericarps are connected by the ventral suture and dorsal suture, and the abscission layer develops only at the ventral suture. The pod pericarp consists of exocarp, mesocarp, and endocarp.

Pods of *M. ruthenica* are composed of the fruit beak, pod pericarp, and fruit stalk (Figure 2), with the pericarps being connected by a ventral and dorsal suture. Furthermore, the ventral suture has an abscission layer. Days 1–20 after flowering correspond to the green maturation stage of pods, days 20–28 after flowering correspond to the yellow maturation stage of pods, and days 28–40 after flowering correspond to the full maturation stage of pods [8]. To clarify whether the two contrasting experimental genotypes have any specific differences in the anatomical structure of the pericarp that might account for their differences in explosive pod shattering occurrence, we analyzed and compared their pod pericarp structures. Specifically, the pod pericarp consists of two layers, an outer (exocarp and mesocarp) and an inner (endocarp) layer. The exocarp is originating from the outer epidermis of the carpel and it is consists of a layer of cells with cuticular wax; these cells are adjacent to the mesocarp cells. The mesocarp comprises four or five layers of parenchyma cells with primary cell walls and large vacuoles. The endocarp is originating from the inner epidermis of the carpel, it is composed of two distinct cell types: two to three layers of cells with lignified cell walls, and a single layer (the transition layer) with less lignified cell walls.

**Figure 3:**
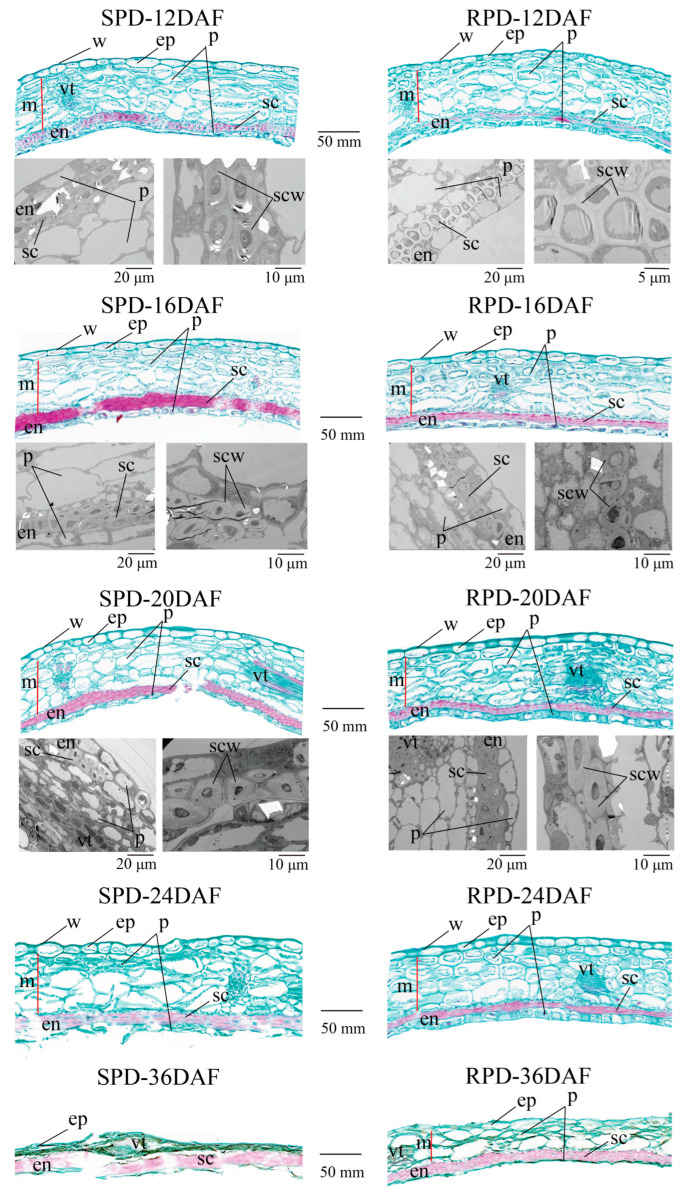
Light microscopy section and TEM section of pod pericarps. Images on the **left** show pod pericarps of SPD, and images on the **right** show pod pericarps of RPD. The **upper** images are saffron-solid green stained pericarp sections observed under an optical microscope; the **lower** images are TEM sections of the endocarp, with a scale of 20 μm (**left**) and 10 μm or 5 μm (**right**), respectively. In these saffron-solid green stained sections, lignified cells are stained red, with deeper red indicating a higher degree of lignification; other structures are stained green. The abbreviations are as follows: ep = exocarp; m = mesocarp; en = endocarp; w = waxy layer; p = parenchymatous cells; vt = vascular tissues; sc = sclereid; scw = secondary cell wall. Compared to RPD, SPD has more layers of sclereids, and from 16 to 20 DAF, the cell walls of these sclereids exhibit well-developed, thick secondary walls with small cell cavities.

Lignification of the sclerenchyma cell layer of the endocarp was observed at 12 DAF (Figure 3 and Figure S1), at which time, the lignification of the SPD endocarp was clearly higher than that observed in RPD, with the sclerenchyma cell walls notably thicker in the first case. Furthermore, the secondary cell wall of SPD sclerotic cells was still developing at 20 DAF, and their cell cavity was narrower than those of RPD sclerotic cells (Table S1). At this time, a uniform accumulation of cell wall materials for a multi-layer sclerenchyma was fairly clearly observed. In addition, we found that cell shrinkage occurred in the mesocarp of SPD at 24 DAF, whereas the mesocarp cells of RPD were still plump. Indeed, the mesocarp of SPD was compressed into a thin layer and became difficult to observe at 36 DAF, while it could still be found in RPD. Finally, we observed a highly lignified endocarp developed in SPD, and cell water loss from the mesocarp. These findings indicate that cell wall-component contents might differ between the two genotypes.

### 3.2. Analysis of Physiological Characteristics Associated with Pod Shattering

**Figure 4:**
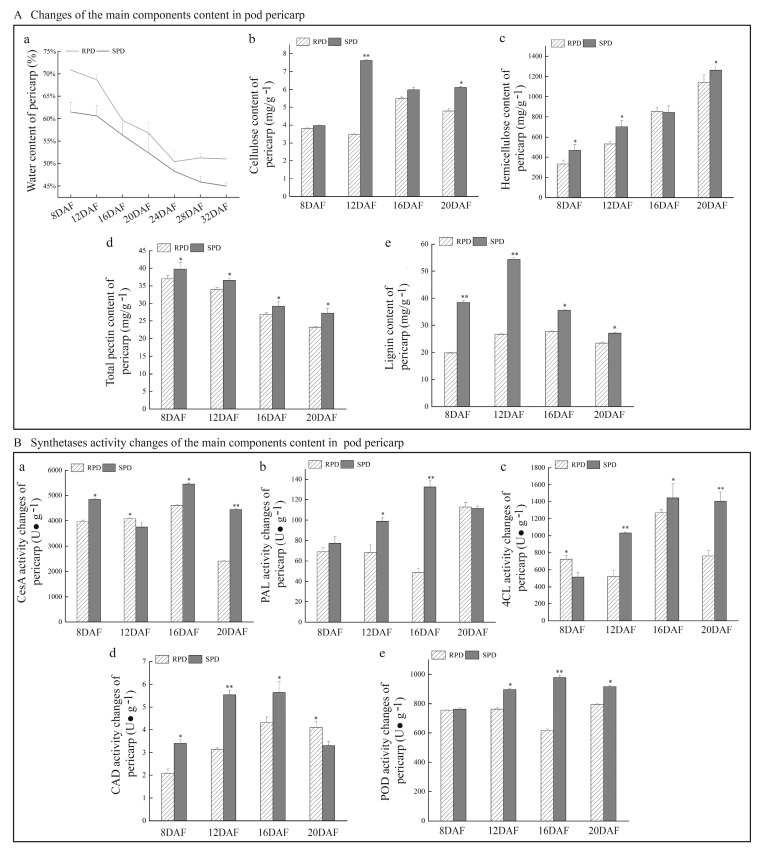
Changes and comparison of physiological characteristics of pod pericarps. Box A represents the changes in main components of pericarp cell wall: the X-axis indicates the pod development stage, and the Y-axis represents the water content (**Aa**), cellulose content (**Ab**), hemicellulose content (**Ac**), total pectin content (**Ad**), and lignin content (**Ae**), respectively. Box B represents the changes in cell wall component-related synthetase activity: X-axis indicates the pod development stage, and Y-axis represents the cellulose synthase (CesA) activity (**Ba**), phenylalanine ammonia-lyase (PAL) activity (**Bb**), 4-coumarate:CoA ligase (4CL) activity (**Bc**), cinnamyl alcohol dehydrogenase (CAD) activity (**Bd**), and peroxidase (POD) activity (**Be**), respectively. The pink line and white striped columns represent RPD, and the green line and gray columns represent SPD. One asterisk represents a significant difference (*p* < 0.05), and two asterisks represent an extremely significant difference (*p* < 0.01). The water content of SPD pod pericarps was lower than that of RPD, but the other contents of cellulose, hemicellulose, pectin, and lignin in SPD pod pericarps were much higher than those of RPD. The CesA activity of SPD pod pericarps was higher than that of RPD during 8DAF, 16DAF and 20DAF. Other enzyme activities of SPD pod pericarps were highest during 16DAF, and it were much higher in SPD than in RPD.

Microstructural observation revealed that the cell walls of the SPD pod pericarp were thicker than those of RPD, and water loss from the pericarp during pod maturity occurred readily. The main components of plant cell walls are cellulose, hemicellulose, and pectin. Lignin is an aromatic compound that accumulates developing a secondary cell wall, strengthening the cell wall as a whole. After determining the contents of the cell wall components in the pod pericarp, we found they were consistent with the observation of dynamic development of the pod pericarp and water content was lower in SPD than in RPD (Figure 4Aa). In particular, at 24 DAF, the water content of SPD decreased from 61.45% to 48.27%, which was lower than that of RPD (50.42%) at that sampling timepoint. Other contents, such as cellulose, hemicellulose, pectin, and lignin, were also much higher in SPD than in RPD, particularly between 12 and 20 DAF. Indeed, only total pectin gradually decreased in the pod pericarp (Figure 4Ad).

The accumulation of plant components is closely related to enzyme activity; therefore, we measured CesA, PAL, 4CL, CAD, and POD activities, to further verify the results of the comparison of cell wall component contents between SPD and RPD. The activities of one cellulose synthase (CesA) and four lignin synthases (PAL, 4CL, CAD, and POD) were higher in SPD than in RPD, particularly over the period from 12 to 16 DAF (Figure 4B).

Principal component analysis was performed to identify the components of the pod pericarp that had the greatest effect on explosive pod shattering. Two principal components were selected according to eigenvalues > 1 (Table 1): eigenvalues of PC_1_ and PC_2_ were 1.819 and 1.305, respectively. The variance contribution rate of PC1 is 45.477%, and that of PC2 is 32.622%, with the cumulative variance contribution rate reaching 78.099%. PC_1_ reflects the characteristics of high-value hemicellulose and low-value pectin, while PC_2_ reflects the synergistic characteristics of high-value lignin and cellulose. Among these, lignin exhibits the highest loading value (0.727), indicating its more prominent influence in pod pericarp.

### 3.3. Analysis of Differentially Expressed Genes (DEGs) and Annotated Pathways Associated with Pod Shattering

The RNA of SPD and RPD was extracted and sequenced using samples collected at 8, 12, 16, and 20 DAF, a total of 24RNA libraries were established and performed a transcriptomic analysis to identify the metabolic pathways and DEGs associated with pod shattering. The RPD group of samples served as control. After filtering, 159.75 Gb reads were obtained. The GC content and Q30 value of these clean data were more than 42.41% and 92.84%, respectively. More than 87.33% of the filtered reads were mapped to the reference genome (Table S2). The number of DEGs in SPD and RPD groups is shown in Figure S2A; a total of 3419 to 7496 DEGs were found at 8 to 20 DAF.

The number of DEGs between the two genotypes increased with pod maturation, with more upregulated DEGs observed at 16 DAF and more downregulated DEGs at 20 DAF. Furthermore, 978 common DEGs were identified across the two genotypes at all four sampling timepoints, while 784, 808, 2524, and 2571 DEGs were specifically expressed at 8, 12, 16, and 20 DAF, respectively (Figure S2B).

To determine the functions of pod shattering-related DEGs, we performed Gene Ontology (GO) enrichment analysis. We identified DEGs corresponding to the following categories: cellular component, molecular function, and biological process (Table S3). Additionally, DEGs were enriched in the following biological processes: carbohydrate metabolic process (GO:0005975), cellular aromatic-compound metabolic process (GO:0006725), and organic-substance metabolic process (GO:0071704). Meanwhile, in the molecular function category, the following were enriched: zinc ion binding (GO:0008270), TF activity, sequence-specific DNA binding (GO:0003700), hydrolase activity (GO:0016787), catalytic activity (GO:0003824), and protein serine/threonine kinase activity (GO:0004674). These categories may be closely associated with pod shattering traits. We subsequently annotated the identified DEGs using KEGG analysis and assigned them to KEGG pathways. The top five metabolic pathways with the significant enrichment and the largest number of DEGs included phenylpropanoid biosynthesis (ko00940, e.g., value at 16 DAF *p* = 4.2 × 10^−309^, with 91 DEGs), plant hormone-signal transduction (ko04075, e.g., value at 16 DAF *p* = 9.8 × 10^−115^, with 194 DEGs), MAPK signaling-pathway plants (ko04016, e.g., value at 16 DAF *p* = 9.8 × 10^−115^, with 145 DEGs), starch and sucrose metabolism (ko00500, e.g., value for 16 DAF *p* = 4.20 × 10^−6^ with 115 DEGs), and plant–pathogen interaction (ko04626, e.g., value at 16 DAF *p* = 9.70 × 10^−6^, with 292 DEGs) (Tables S4–S8). These results suggest that the developmental stages of the pod pericarp were consistent, particularly phenylpropanoid biosynthesis, which is in agreement with the anatomical structure and physiological data.

**Figure 5:**
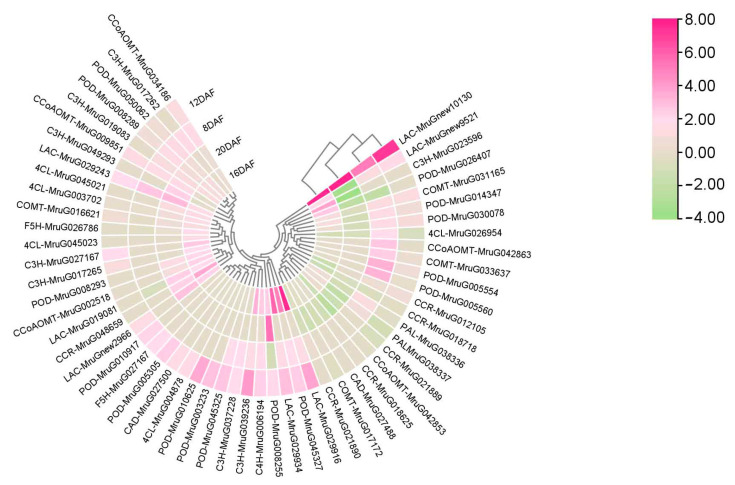
Heat maps of DEGs involved in the phenylpropanoid biosynthesis pathway. Each row represents one gene ID; genes with the same function are marked with the same color bar along the left edge of the heat map. Levels of gene expression are represented by a color gradient ranging from blue (downregulated) to white (non-significance) to red (upregulated).

As highly lignified, thick, secondary cell walls were found in the endocarp in SPD, which may have led to the pod shattering trait, we performed an analysis of phenylpropanoid biosynthesis, which is derived from the shikimate pathway responsible for the synthesis of lignins, flavonoids, and auxin. Genes involved in phenylpropanoid biosynthesis for lignin production were upregulated at 12–16 DAF in pod pericarp. Most DEGs were upregulated in the SPD group, in which case, they were twice as many as those upregulated in the RPD group (Table S9). A total of 57 DEGs were identified (Figure 5), including 2 *PAL*s, 1 *C4H*s, 8 *C3H*, 5 *4CL*, 5 *CCoAOMT*, 2 *F5H*, 4 *COMT*, 6 *CCR*, 2 *CAD*, 15 *POD*, and 7 *LAC* genes. Among these DEGs, the upregulated ones were concentrated downstream of lignin production (*POD*s and *LAC*s), especially in the lignin monomer-polymerization assembly. The expression of MruG006194 (*C4H*), MruG039236 (*C3H*), MruG031165 (*COMT*), MruG045327 (*POD*), MruG08255 (*POD*), MruGnew9521 (*LAC*), MruGnew10130 (*LAC*), MruG029243 (*LAC*), MruG029916 (*LAC*), and MruG029934 (*LAC*) remained upregulated from 8 to 16 DAF. Subsequently, we selected genes for transcriptome data validation by qRT-PCR and found that the R^2^ value reached 0.88, implying that our transcriptomic data were reliable (Figure S3, Table S10).

### 3.4. Possible Upstream Regulators of POD Gene Expression

Transcription factors regulate gene expression by binding to specific DNA sequences, such as cis-regulatory elements in the promoter region of their target genes. The *POD*s that encode peroxidase are essential for lignin polymerization, and the number of *POD* DEGs were greater than other function genes in phenylpropanoid biosynthesis pathway. Thus, the promoter analysis of *POD*s was performed using PlantCARE; therefore, 2000 bp before the start codon were identified among 13 *POD* sequences (Table S11). The analysis revealed that the promoters of 11 *POD*s contained *MYB*-binding motifs, while 8 *POD*s contained auxin-responsive elements (AuxRR-core or TGA-element, Table S11). These findings indicated that *POD*s might be directly bound by *MYB* TFs and interact with the auxin signaling pathway.

Therefore, we further analyzed the expression level of *MYB* TF family members in the two genotypes and found that 76 *MYB* TFs showed a high number of transcripts in the SPD genotype (Table S12). Although most *MYB*s showed the largest number of DEGs at 16 and 20 DAF, only MruG025571 (*MYB*-like protein X) was upregulated at four pod developmental points, and MruG039026 (*MYB*61) was upregulated from 8 to 16 DAF. Conversely, MruG039168 (*MYB*/*SANT* family) and MruG043723 (*MYB*306) were downregulated at four pod developmental points.

With respect to auxin, we identified nine DEGs that function in relation to auxin synthase, including *TAA* (tryptophan aminotransferase, which converts tryptophan to indole-3-pyruvate (IPA)), *YUC* (indole-3-pyruvate monooxygenase, which converts IPA to IAA) and FMO (flavin-containing monooxygenases, coded by *YUCCA* involved in tryptophan-dependent auxin synthesis).

Of these nine DEGs, the majority were downregulated in the SPD genotype (Table S12), with one DEG upregulated at 8 DAF and two DEGs upregulated at 20 DAF. Only MruG026138 (*YUC*/*FMO*) was downregulated from 8 to 20 DAF. These findings suggest that pod shattering in *M. ruthenica* is negatively regulated by auxin.

**Figure 6:**
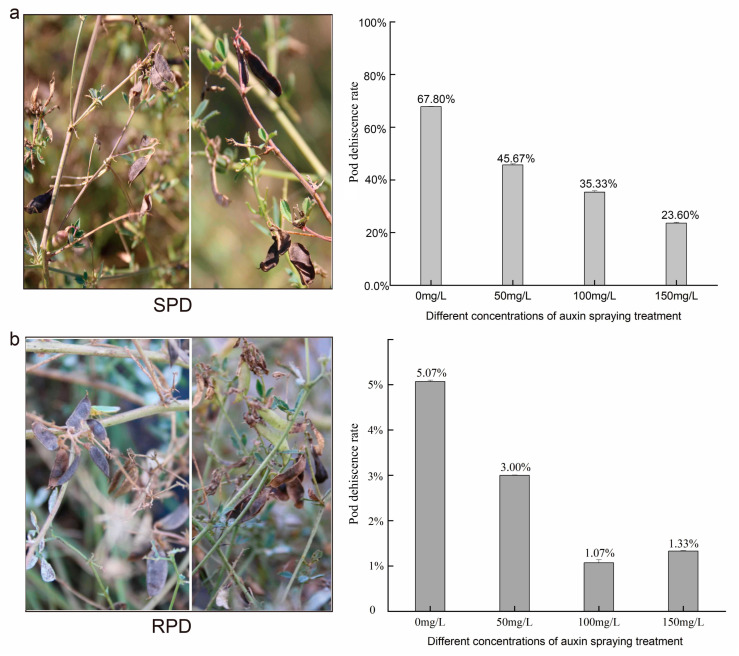
Verification of 4-CPA spraying on pods of SPD and RPD. (**a**): changes in pods of SPD in the field on the left, 4-CPA at different dilution levels effect on pod shattering rate of SPD was showed on the right, the X axis represents different concentrations of 4CPA, and the Y axis represents the pod shattering rate. (**b**): changes in pods of RPD in the field on the left, 4-CPA at different dilution levels effect on pod shattering rate of RPD was showed on the right, the X axis represents different concentrations of 4CPA, and the Y axis represents the pod shattering rate.

The further validation of 4-CPA application showed that the pod shattering rate decreased from 67.8% to 23.6% in SPD plants (Figure 6), whereas in RPD plants it decreased from 5.07% to 1.07%. Furthermore, those pods of RPD delayed maturation, even becoming increasingly slender. We subsequently analyzed the expression levels of *POD*s using RT-qPCR (Figure S4), and found that the expression levels of most *POD*s in treated groups (C50 to C150) were lower than those in the control group (C0). This result indicated that auxin negatively regulated pod shattering and *POD* expression in *M. ruthenica*.

## 4. Discussion

### 4.1. Increased Lignin Content Altered the Properties of the Pericarp Material and Promoted Complete Pod Shattering

For several decades, studies have focused on exploring the pod shattering mechanism across multiple economic crops or model plants, particularly in the abscission zone associated with pod shattering-resistant traits [8,30,31]. High levels of polygalacturonase (PG) and cellulase (CE) activities cause the cells in the abscission zone to break down, leading to pod shattering in SPD *M. ruthenica*. In contrast, lower levels of PG and CE activities result in tightly closed pods [8]. However, the reason for the presence of pods with slit shattering in RPD *M. ruthenica* remains unclear. We observed that the cells in the abscission zone broke down and pod pericarps were separated from each other (Figure 1) [8]. This suggests that the destruction of abscission zone cells by PG and CE activities is not sufficient for *M. ruthenica* pods to open and release their seeds rapidly [8,32,33], a mechanical force is also required to cause strain on the pericarps [34].

The force required for the explosive opening of pods is typically generated by the tension created by pericarp dehydration [34]. Indeed, we observed cellular dehydration and shrinkage distortion occurring in the mesocarp of SPD plants at 24 DAF (Figure 3). Further, the water content of the pericarps decreased as pods matured (Figure 4). As the pods desiccate, water loss and shrinkage of the outer layers of the pericarp (exocarp and outer mesocarp) likely generate a considerable tension force on the rigid inner layer (endocarp), which tends to pull the two pericarps away from each other; thus, when water loss from the inner layer begins, little force is generated for pod shattering, which may tend to push the two pericarps close to each other [35]. The lignified pericarp is similar to that of wood. According to wood mechanics, this force actually named dry shrinkage stress [36]. Drying typically causes woods shrinkage, which often leading to internal stresses reduction and potential dehiscence or warping, as well as its stiffness, flexural strength and impact toughness reduction [37,38,39,40,41]. Once the pericarp completes the drying process, the pericarp becomes warped, and its flexural strength and impact toughness decline to facilitate pod dehiscence [39,40,41]. The small size of *M. ruthenica* pods makes it difficult to determine the corresponding pod shattering force involved, or to calculate the corresponding pod shattering power. However, the formation and magnitude of the material force are closely related to the properties of the material itself, such that we can elucidate the mechanism underlying *M. ruthenica* pod explosive shattering from the inferred properties of pod pericarps.

It has also been reported that the tension of explosive pod shatter is controlled by lignin deposition, one of the principal components closely related to pod shattering (Figure 4, Table 1), within the endocarp [40]. Consistent with this reasoning, we found that the lignin content and related synthetase activities were higher in SPD than in RPD plants. Similarly to the legume forage of common vetch (*Vicia sativa*), the total lignin content of shattering-susceptible accessions was significantly higher than that of the shattering-resistant accessions [42]. Lignin is a hydrophobic phenolic compound, and an increase in lignin content can significantly affect the hygroscopicity, water content, and water retention capacity of a given material [43,44,45,46]. Thus, lignin accumulation can lead to the characteristics of SPD pod pericarps, with lower water content, rapid water loss, and extremely poor water retention capacity. Pod shattering of *Phaseolus vulgaris* is also correlated with high lignin content in the pod pericarps [47]. However, leguminous species are clearly distinct from gramineous species, such as *Oryza sativa* or *Elymus sibiricus*, where seed shattering can be induced by repressing lignin deposition [48,49]. In contrast, in leguminous species, high lignin content and hydrolase activity are necessary for explosive pod shattering. In this case, high levels of lignin accumulation change the physical properties of the pod pericarp, and hydrolase activity in the pod ventral suture disrupts the adhesion of the two pericarps.

### 4.2. Auxin Negatively Regulates Phenylpropanoid Biosynthesis and the Explosive Opening of M. ruthenica Pods

Lignin is the final product of phenylpropanoid biosynthesis. Consistent with the results reported for *Phaseolus vulgaris* [47], in this study, metabolic events associated with pod shattering in *M. ruthenica* were related to phenylpropanoid biosynthesis (Table S4). Furthermore, upregulated DEGs involved in the lignin biosynthesis pathway, including *POD*s and *LAC*s, which control and polymerize lignin monomers into complex phenolic polymers [50,51], were mainly present downstream of the lignin monomer-polymerization assembly. In line with these findings, transcriptome analysis of *Arabidopsis thaliana* showed that peroxidase genes were potentially involved in pod shattering [52]. From this perspective, the major difference between SPD and RPD in pod physical capability for explosive shattering likely relates to lignin monomer polymerization.

Notably, we found that the *POD*s promoter region contained auxin cis-regulatory elements (Table S11), whereas DEGs related to auxin biosynthesis were downregulated (Table S12). Another study on plant hormone-metabolome determination of *M. ruthenica* pods revealed lower auxin contents in SPD [53]. This finding indicates that lignin biosynthesis might be negatively regulated by auxin. In *Arabidopsis* pods, studies on transgenic lines of the *IND* gene revealed that a minimum auxin level is required for the development of endocarp lignification. These transgenic lines exhibit increased auxin content, a lack of lignified endocarp layers, and indehiscent pods [54]. Studies on secondary cell wall development in *Arabidopsis* indicated that auxins negatively induced secondary cell wall formation [55]. This is possible because both auxin synthesis and phenylpropanoid biosynthesis begin with the shikimic acid pathway, which shares the same precursor substrates, leading to competition and inhibition [24,56,57]. Coincidentally, genes encoding PG and CE in the SPD genotype of *M. ruthenica* were also found to be negatively regulated by auxin [8], indicating that complete pod shattering in *M. ruthenica* is negatively regulated by auxin. Moreover, exogenous auxin application to the SPD genotype reduced its pod shattering rate (Figure 6), suggesting a management practice at harvest for controlling seed losses due to pod shattering.

## 5. Conclusions

In this study, microstructural, physiological, and transcriptome analyses of *M. ruthenica* pod pericarp were conducted to investigate potential factors in pod shattering. Observations suggest an association between pod shattering in this species and high lignification of the pod endocarp, along with increased lignin content. Transcriptome analysis identified over 3419 DEGs, with KEGG enrichment indicating phenylpropanoid biosynthesis (ko00940) as the top pathway. Within this pathway, 57 functional genes were selected, 15 of which were annotated as peroxidases (*POD*s). Bioinformatic analysis predicted that most of these *POD*s contain *MYB*-binding motifs and auxin-responsive elements in their promoters. Concurrently, most MYB transcription factors were upregulated in the SPD genotype, while most auxin biosynthesis genes were downregulated. Critically, exogenous auxin supplementation experimentally demonstrated a negative correlation of auxin with both *POD*s expression and pod shattering rate in SPD. Collectively, these findings provide preliminary insights and may serve as candidate genetic resources for future breeding efforts in *M. ruthenica* and related legume crops.

## Figures and Tables

**Table 1:** Principal component analysis of cell wall components associated with pod shattering.

Principal Component	Eigenvalue	Percentage of Variance (%)	Cumulative (%)	Eigen Vectors			
				Pec	Cel	Hec	Lig
PC_1_	1.819	45.477	45.477	−0.623	0.358	0.688	−0.106
PC_2_	1.305	32.622	78.099	0.316	0.603	0.085	0.727
PC_3_	0.642	16.061	94.160	0.329	0.652	−0.144	−0.668

Cel = cellulose content; Hec = hemicellulose content; Pec = pectin content; Lig = lignin content. PC1 to 3, principal component 1 to 3.

## Data Availability

The original contributions presented in this study are included in the article. Further inquiries can be directed to the corresponding author.

## Supplementary Materials

The following supporting information can be downloaded at: https://www.mdpi.com/article/10.3390/biom15091269/s1, Figure S1: Microscopic anatomical figures of the pod pericarp dynamic development; Figure S2: Analysis of DEG expression in the SPD and RPD at different stages; Figure S3: Correlations between RNA-Seq and RT-qPCR results of phenylpropanoid biosynthesis at different sampling time points; Figure S4: Heat map of the PODs expression in SPD with different auxin concentration traits; Table S1: Quantitative measurements analysis by ImageJ of endocarp cells; Table S2: Sequencing data statistics and analysis of *M. ruthenica* pod pericarps; Table S3: GO annotation of differentially expressed genes; Table S4: KEGG pathway annotation of differentially expressed genes; Table S5: Differentially expressed genes at 8 d after flowering (DAF); Table S6: Differentially expressed genes at 12 d after flowering (DAF); Table S7: Differentially expressed genes at 16 d after flowering (DAF); Table S8: Differentially expressed genes at 20 d after flowering (DAF); Table S9: Expression levels of 57 genes involving in phenylpropanoid biosynthesis; Table S10: Primers used for RT-qPCR and expression levels; Table S11: Promoter analysis of POD encoding genes; Table S12: Expression levels of MYB TFs and auxin synthase.
