# Supplementary material for: Anatomical, Physiological, and Transcriptome Analyses Revealing Pod Shattering of Medicago ruthenica Associated with Pericarp Lignin Biosynthesis
Source: Biomolecules. 2025 Sep 2;15(9):1269. doi: 10.3390/biom15091269 (PMC12467310; doi:10.3390/biom15091269)
Supplement: Supplementary file 1 [file biomolecules-15-01269-s001.zip › Supplementary Figures.doc]

**Figure S1** Microscopic anatomical figures of the pod pericarp dynamic development.

Note: Images on the left show pod pericarps of SPD, and images on the right show pod pericarps of RPD. In these saffron-solid green stained sections, lignified cells are stained red, with deeper red indicating a higher degree of lignification; other structures are stained green. The abbreviations are as follows: ep = exocarp; m = mesocarp; en = endocarp; w = waxy layer; p = parenchymatous cells; vt = vascular tissues; sc = sclereid; scw = secondary cell wall. Compared to RPD, SPD has more layers of sclereids, and from 16 to 20 DAF, the cell walls of these sclereids exhibit well-developed, thick secondary walls with small cell cavities.

**Figure S2 Analysis of DEG expression in the SPD and RPD at different stages.**

Note: A: Column chart displaying the differentially expressed gene (DEG) number at the four pod development stages. B. Venn plot describing the distribution of DEGs in the four pod development stages.

**Figure S3 Correlations between RNA-Seq and RT-qPCR results of phenylpropanoid biosynthesis at different sampling time points.**

The correlation between transcriptome data and RT-qPCR data was determined by expression fold changes. As shown in Figure S2, a scatter plot is generated to compare the discounted changes. Pearson correlation coefficient ( R^2^ ) was calculated to evaluate the linear regression between RT-qPCR and transcriptome data. The R^2^ value of these genes was 0.88, the expression of these genes determined by qRT-PCR were consistent with the transcriptome data. It indicates that our transcriptome data are reliable.

**Figure S4 Heat map of the *POD*s expression in SPD with different auxin concentration traits.**

Note: A heatmap was drawn with TBtools. Gene expression were calculated by 2^−ΔΔCT^ method are color coded, from red (highly expressed) to white (appropriately expressed) to blue (low expressed), deeper color variations represent greater differences in relative gene expression). Each line represent related gene ID. C0, C50, C100 and C150 represent 0mg / L 4-CPA, 50mg / L 4-CPA, 100mg / L 4-CPA, 150mg / L 4-CPA, respectively. Each column represent sampling times of 8, 12, 16, 20 DAF. As shown in the figure, exogenous spraying auxin can down-regulated POD expression.
